# Detection of Crimean-Congo Haemorrhagic Fever cases in a severe undifferentiated febrile illness outbreak in the Federal Republic of Sudan: A retrospective epidemiological and diagnostic cohort study

**DOI:** 10.1371/journal.pntd.0007571

**Published:** 2019-07-10

**Authors:** Hilary Bower, Mubarak El Karsany, Mazza Alzain, Benedict Gannon, Rehab Mohamed, Iman Mahmoud, Mawahib Eldegail, Rihab Taha, Abdalla Osman, Salim Mohamednour, Amanda Semper, Barry Atkinson, Daniel Carter, Stuart Dowall, Jenna Furneaux, Victoria Graham, Jack Mellors, Jane Osborne, Steven T. Pullan, Gillian S. Slack, Tim Brooks, Roger Hewson, Nicholas J. Beeching, Jimmy Whitworth, Daniel G. Bausch, Tom E. Fletcher

**Affiliations:** 1 UK Public Health Rapid Support Team, London, United Kingdom; 2 Department of Infectious Disease Epidemiology, London School of Hygiene & Tropical Medicine, London, United Kingdom; 3 Department of Medical Microbiology, Karary University, Khartoum, Sudan; 4 National Public Health Laboratory, Federal Ministry of Health, Khartoum, Sudan; 5 Communicable Disease Surveillance and Event Unit, Federal Ministry of Health, Khartoum, Sudan; 6 Global Public Health, Public Health England, London, United Kingdom; 7 National Infection Service, Public Health England, Porton, United Kingdom; 8 Clinical Sciences, Liverpool School of Tropical Medicine, Liverpool, United Kingdom; 9 Department of Disease Control, London School of Hygiene & Tropical Medicine, London, United Kingdom; Naval Medical Research Center; Biological Defense Research Directorate, UNITED STATES

## Abstract

**Background:**

Undifferentiated febrile illness (UFI) is one of the most common reasons for people seeking healthcare in low-income countries. While illness and death due to specific infections such as malaria are often well-quantified, others are frequently uncounted and their impact underappreciated. A number of high consequence infectious diseases, including Ebola virus, are endemic or epidemic in the Federal Republic of Sudan which has experienced at least 12 UFI outbreaks, frequently associated with haemorrhage and high case fatality rates (CFR), since 2012. One of these occurred in Darfur in 2015/2016 with 594 cases and 108 deaths (CFR 18.2%). The aetiology of these outbreaks remains unknown.

**Methodology/Principal findings:**

We report a retrospective cohort study of the 2015/2016 Darfur outbreak, using a subset of 65 of 263 outbreak samples received by the National Public Health Laboratory which met selection criteria of sufficient sample volume and epidemiological data. Clinical features included fever (95.8%), bleeding (95.7%), headache (51.6%) and arthralgia (42.2%). No epidemiological patterns indicative of person-to-person transmission or health-worker cases were reported. Samples were tested at the Public Health England Rare and Imported Pathogens Laboratory using a bespoke panel of likely pathogens including haemorrhagic fever viruses, arboviruses and *Rickettsia*, *Leptospira* and *Borrelia* spp. Seven (11%) were positive for Crimean-Congo haemorrhagic fever virus (CCHFV) by real-time reverse transcription PCR. The remaining samples tested negative on all assays.

**Conclusions/Significance:**

CCHFV is an important cause of fever and haemorrhage in Darfur, but not the sole major source of UFI outbreaks in Sudan. Prospective studies are needed to explore other aetiologies, including novel pathogens. The presence of CCHFV has critical infection, prevention and control as well as clinical implications for future response. Our study reinforces the need to boost surveillance, lab and investigative capacity to underpin effective response, and for local and international health security.

## Author summary

The Federal Republic of Sudan has had at least 12 outbreaks of febrile illness of unknown cause associated with symptoms of haemorrhage and high case fatality rates since 2012. Outbreaks without clear diagnosis are concerning, particularly in countries such as Sudan where a range of high consequence diseases, including viral haemorrhagic fevers, are endemic or epidemic, and local laboratory capacity is limited. We transferred historical samples stored in the National Public Health Authority from one of these outbreaks that occurred in Darfur 2015–2016 to the Public Health England Laboratory at Porton, UK, and tested them against a wide range of infectious diseases to try to identify the cause, and to help the Sudanese Federal Ministry of Health to develop and target their limited laboratory capacity. We found that Crimean-Congo Haemorrhagic Fever was an important cause but not the only source of cases in this outbreak. This has implications for prevention and control as well as for treating cases. Our study also highlighted the need for future studies to explore other possible causes, including new pathogens, and reinforced the need to boost surveillance, lab and investigative capacity for more timely and complete outbreak response.

## Introduction

Undifferentiated febrile illness (UFI) is one of the most common reasons for people seeking healthcare in many low-income countries.[1] While illness and death due to some specific infections such as malaria are often well-quantified, others which can be caused by a wide range of pathogens, are frequently uncounted and their impact therefore underestimated.[2,3] At least 12 outbreaks of undifferentiated febrile illness have been reported in the Federal Republic of Sudan since 2012, frequently associated with haemorrhage and high case fatality rates, including a cluster in the Darfur region of Sudan in 2015/2016 which resulted in 594 cases and 108 deaths over 27 localities (Table 1). The aetiology of these outbreaks remains unknown.

**Table 1:** Outbreaks of fever with haemorrhagic symptoms reported by the Federal Ministry of Health and in ProMED-mail, in the Republics of Sudan & South Sudan, 2012–2017.

**SUDAN**				
**Month/Year**	**Location affected**	**Main symptoms /suspected cause**	**Cases**	**Deaths (%)**
Sept 2012 [4]	Zalingei, Central Darfur	Headache, vomiting, fever	unkn	7
Oct 2012 [4]	Korley, South Darfur	Fever	16	6 (37.5)
Dec 2012- Oct 2013 [5–7]	Greater Darfur	Several syndromes: yellow skin, vomiting and diarrhoea; face/leg swelling, rash leading to bruising, mainly children. Considered to be yellow fever	847	171 (20.2)
Jun 2014 [8]	Kalma, South Darfur	High fever, loss of appetite, headache, pain in chest, vomiting	unkn	18
Oct 2014 [9]	El Fula, Muglad, West Kordofan	High fever, bleeding, nosebleed	4	4 (100.0)
Nov 2014 [10]	Kalma, South Darfur	Fever, headache, high fever, bleeding (nose/mouth)	unkn	3
Nov 2014 [11]	South Kordofan	High fever, vomiting, headache. ?Haemorrhagic fever and/or kala azar—unconfirmed	25	unkn
Nov 2014 [12]	Wad Madani, El Gazeera	Fever, bleeding: suspected haemorrhagic fever	30	19 (63.0)
Aug 2015 –Apr 2016 [13,14]	Greater Darfur	Fever, bleeding (98%), joint pain, jaundice. ?Dengue	594	108 (18.2)
Oct 2015—Feb 2016	Kordofan	?Dengue (6 lab +ve), no profuse bleeding	47	4 (8.5)
Oct 2015—Feb 2016	Kassala	?Dengue (4 ELISA IgM +ve)	7	1 (14.3)
Apr–Nov 2017 [15]	Tokar, Red Sea State	Fever and “haemorrhagic” symptoms	82	unkn
**SOUTH SUDAN**				
2003 [16]	Imatong, Equatoria	Headache, fever, bloody diarrhoea, nosebleeds	178	11 (6.2)
Sept 2013[17]	Pibor, Jonglei	Fever, headache, paralysis: rapidly fatal.	unkn	15
Dec-2015—May 2016 [18]	Aweil North & West	Fever, bleeding, fatigue, headache, vomiting. 33 samples tested externally: 5 positive for o’nyong ‘nyong; 3 for chikungunya, 1 dengue. All negative for viral haemorrhagic fever. ?Susugo paramyxovirus ?Lujo virus.	55	10 (18.2)

‘?’ denotes postulated cause of outbreak without confirmation or with inadequate confirmed cases to be considered responsible for the outbreak. Grey highlighted row is the outbreak investigated in this study. Bracketed numbers refer to entries in the reference list.

A range of high consequence infectious diseases (HCID) are endemic or epidemic in Sudan including Ebola virus, which was identified contemporaneously in Sudan and the Democratic Republic of Congo in 1976. [19,20] The National Public Health Laboratory (NPHL) of Sudan intermittently identifies viral haemorrhagic fever (VHF) cases including Rift Valley fever (RVF) and Crimean-Congo haemorrhagic fever (CCHF). Sudan is also at high risk of yellow fever virus (YFV) epidemics, the most recent outbreak occurring in 2012/13.[21] Outbreaks of dengue fever and chikungunya are common, and cases of West Nile fever have been recorded. (Table A in S1 Text). There have also been outbreaks of undiagnosed fever with haemorrhagic symptoms in South Sudan, most recently in Aweil State in 2016, and of RVF in Eastern Lakes State in 2018.

Outbreak investigation in Sudan is the responsibility of the ministries of health of the 18 states, reinforced by the Federal Ministry of Health (FMoH) when cases appear to increase significantly or exhibit characteristics of HCID and/or person-to-person transmission. Alerts are received through 1659 sentinel sites (27% of all health facilities) which transmit reports weekly or daily depending on the disease. In past outbreaks, epidemiological data have often been incomplete and laboratory analysis delayed due to the limited capacity of state-level laboratories which necessitates transfer of samples for analysis to the NPHL in Khartoum. Rapid Response Teams comprising epidemiology, laboratory and clinical staff have been established in each State and at Federal level to improve the speed and depth of outbreak investigation.

The Darfur outbreak, reported to FMoH on September 21, 2015 and eventually affecting all 5 states in the Darfur region, was initially thought to result from severe dengue complicated by malaria and other underlying conditions such as sickle cell disease commonly found among some tribes in Darfur.[22] However, only 24% of the suspect case samples received and tested by NPHL showed evidence of dengue virus (DENV) IgM antibodies, and no samples were positive by PCR. Samples tested for viral haemorrhagic fevers at the WHO collaborating laboratory in Dakar, Senegal and the Robert Koch Institute in Berlin were also all negative. Limited in their capacity to test further due to restrictions on reagent importation, the NPHL tested a small number of samples (30) in-house for CCHFV and found 7 were PCR positive, all from East Darfur. A further 7 samples were found to have serological evidence of West Nile fever virus but as neutralisation tests were not available, a cross-reaction could not be ruled out.

**Fig 1:**
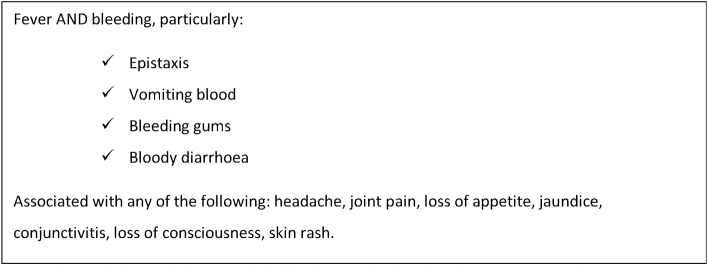
Case definition used in the field during the Darfur outbreak.

**Fig 2:**
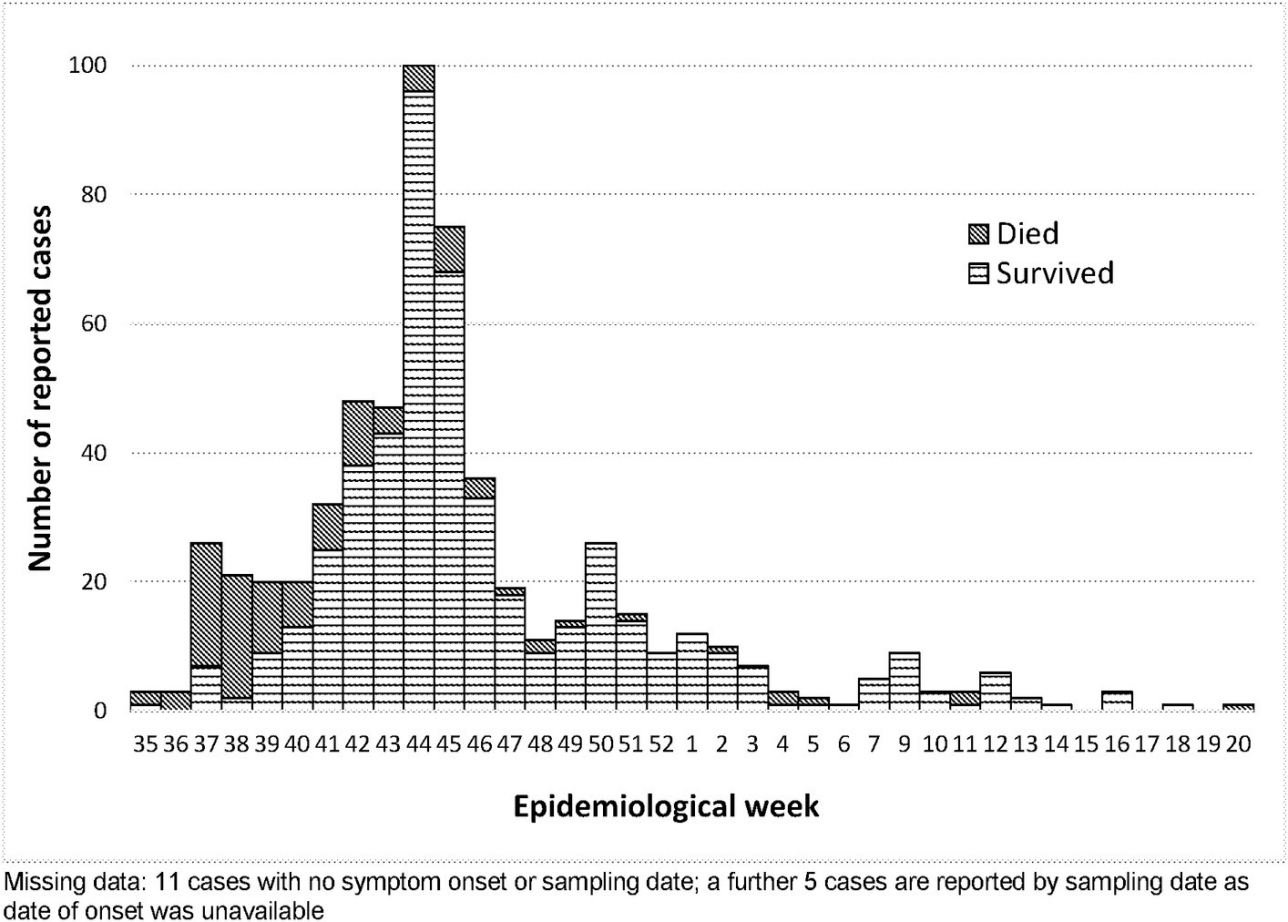
Reported cases and deaths, August 24, 2015—May 9, 2016 by symptom onset (n = 594).

Over the next 9 months, 594 cases were reported using the outbreak case definition (Fig 1) across 27 localities of Darfur, the majority coming from West Darfur and focused on Kereinik, followed by Al Sareef in North Darfur, and Zalingei in Central Darfur–areas which hosted substantial numbers of displaced people. Cases peaked in November 2015 and dropped to sporadic cases from February until the announced end of the outbreak in May 2016. (Fig 2) Since then, at least 3 smaller outbreaks of a similar syndrome have occurred in different locations in Sudan.

The unknown source(s) and ill-defined characteristics of these outbreaks are of significant concern. To try to identify the outbreak pathogen(s), better characterise the outbreak syndrome, inform public health and treatment interventions, and assist development of diagnostic capacity, we investigated a set of stored samples from the outbreak with a bespoke panel of molecular and serological assays and epidemiological analysis.

## Materials and methods

### Ethics statement

Approval for the study was granted by the FMoH Technical Review Board, and the Ethics Committees of Karary University, Khartoum and the London School of Hygiene & Tropical Medicine (Ref: 11930). Individual consent was not required as samples were collected for diagnostic purposes during the public health response to the outbreak. Permission to export samples to the UK was given by the FMoH and samples and data were anonymised before transfer.

**Fig 3:**
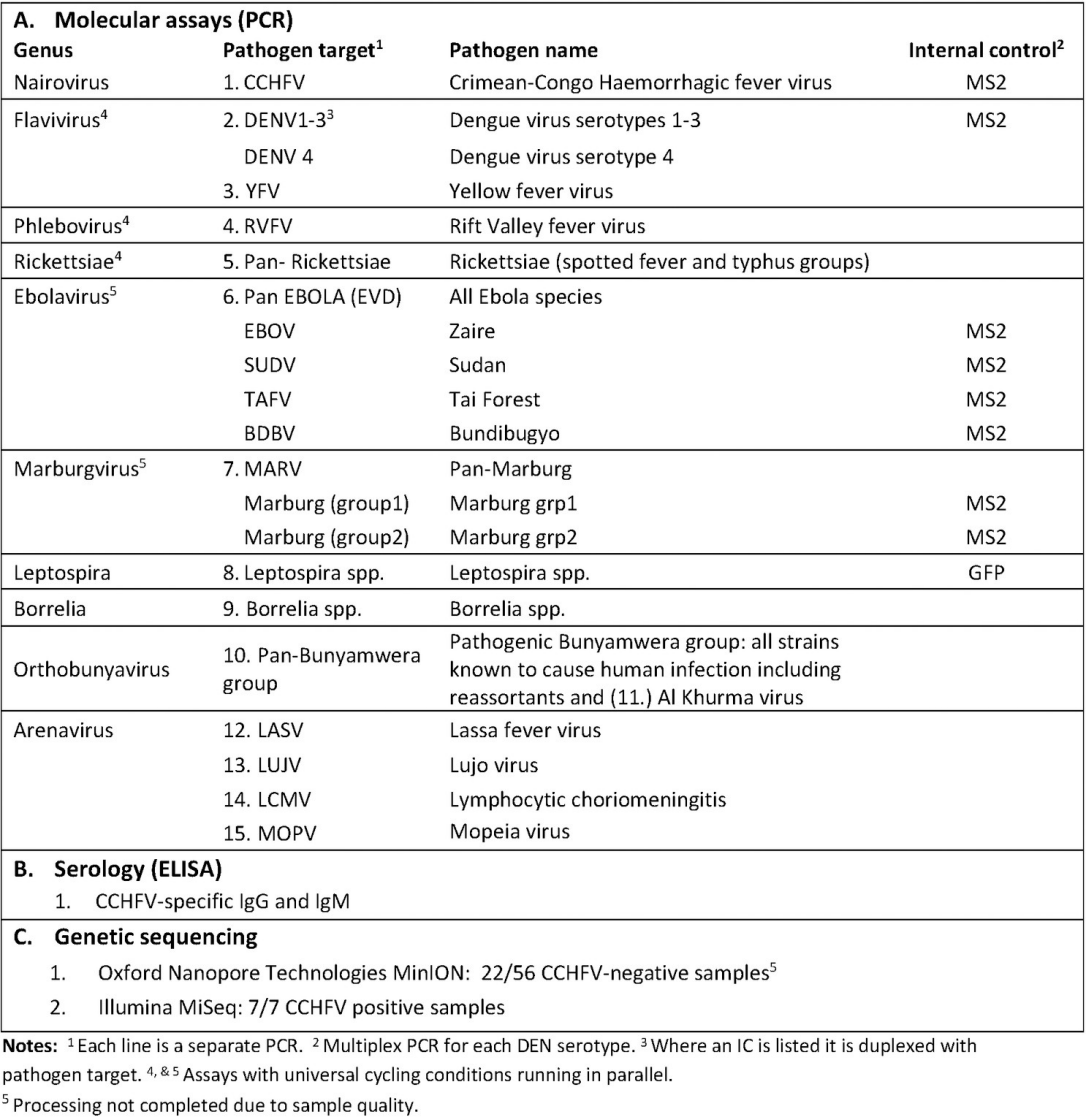
Assays and sequence of testing of transferred samples.

The NPHL received blood samples from 263 of the 594 cases notified during the outbreak, from which serum was stored. After matching data held by the FMoH Epidemiology Unit with samples and data stored at the NPHL, we evaluated samples for inclusion in the study using the following criteria: sufficient sample volume to accommodate multiple tests (>300 μl); sufficient epidemiological data to allow minimum characterisation of the case; specimen not previously tested by an external laboratory, and not previously tested positive for CCHFV in NPHL. Sixty-five (65) of the 263 stored samples met the criteria and were transported as frozen serum to the Rare and Imported Pathogens Laboratory (RIPL), Porton, UK where assays covering a broad range of likely pathogens were performed (Fig 3). As sample volume was limited, PCR assays were chosen based on clinical syndrome and geographical location and included haemorrhagic fever viruses, arboviruses, arenaviruses, leptospirosis and rickettsiae. Samples that did not meet the criteria (198) were not transferred and remained stored in NPHL. Sensitivity analysis was performed to assess if the samples transferred to the UK were representative of the samples that remained in Sudan.

### Laboratory methods

Detailed information on the design and conditions for each assay is available in the Technical Appendix: Laboratory and Metagenomic Materials and Methods in Supporting Information (S1 Text)

#### Molecular analysis

PCRs using nucleic acid extracted from plasma were performed on all 65 samples in parallel, for pathogens and in the sequence shown in Fig 3, and in accordance with RIPL clinical protocols. After the detection of CCHF RNA, serological assays for CCHF IgG and IgM were performed on all 65 samples to investigate if acute cases were present that had not been identified by PCR and if there was evidence of previous exposure.

#### Serological analysis

Plasma samples in which CCHFV RNA was detected were transferred to a containment level (CL) 4 laboratory in PHE Porton and heat-inactivated at 56°C for 30 minutes (with intermittent sample-mixing) before transfer to the CL2 laboratory in the same location for ELISA analysis. Samples in which no CCHFV RNA was detected were analysed without additional pre-treatment. VectoCrimea-CHF ELISA kits (Vector-Best, Novosibirsk, Russia) were used for detection of CCHFV-specific IgG and IgM. Absorbances were read at an optical density of 450nm (OD450_nm_) on a microplate reader (SpectraMax M3, Molecular Devices, UK) and analysed using SoftMax Pro (version 7.0, Molecular Devices, UK). The cut-off value was calculated as the average OD_450nm_ of the negative controls supplied with the kit + 0.2. Samples were considered to be positive if their OD_450nm_ was greater than or equal to this cut-off value.

#### Genome sequencing

A metagenomic RNA sequencing protocol and analysis, described in detail previously, was applied to fresh extracts of the 7 CCHFV positive samples and sequenced in the RIPL on a MiSeq (Illumina, UK).[23] CCHF-negative samples were also prepared for metagenomic sequencing using residual extracts remaining after PCR as too few had primary sample available for fresh extract. However, processing of this group was halted after analysis of 22/58 revealed inadequate RNA quality.

#### Data analysis

Data cleaning and analysis was carried out at the London School of Hygiene & Tropical Medicine (LSHTM) in collaboration with the FMoH epidemiology team. Results are expressed as the median and Inter-Quartile Range (IQR) for continuous variables, and frequencies and percentages (n (%)) for categorical variables. Associations were tested using Fisher’s exact test or Chi^2^ as appropriate. We compared the characteristics of the samples transferred to RIPL with those that were not transferred for potential bias and assessed the effect of missing values. Variables without a definitive entry (e.g. yes/no, positive/negative) were counted as missing data. Significance level was set at <0.05. All analyses were completed in STATA (StataCorp LLC, USA, V14.2).

## Results

Forty-three per cent (263) of the 594 cases notified in the outbreak had samples sent to NPHL. The majority of those sampled came from West Darfur (40.3%, 104/258), Central Darfur (23.6%, 61/258), and East Darfur (15.5%, 40/258), with the remainder from North and South Darfur (55/258) and 5 samples without location data.

The majority of all sampled cases were children or young adults and were either at school or studying or working in farming or animal husbandry. There were no samples from healthcare workers. The most common symptoms among all sampled cases were fever (95.8%, 252/263)), bleeding (95.7%, 235/243), mainly epistaxis (57.4%, 140/244) and hematemesis (53.5%, 129/241) headache (51.6%, 128/248) and arthralgia (42.2%, 103/244) (Table 2).

**Table 2:** Sensitivity analysis of characteristics of samples from the Darfur outbreak, 2015–2016 tested in RIPL (n = 65) and samples not meeting testing criteria (n = 198).

Total outbreak case samples received by NPHL (N = 263)	Samples remaining in NPHL after selection criteria applied % (cases/n—missing data)	Samples meeting criteria & transferred & tested in RIPL % (cases/n—missing data)
**Sex** (Male)	64.1% (127/198)	51.6% (33/64)
**Age**: median (IQR) (n)	13.5 (7–25) years (198/198)	14.0 (9–25) years (61/65)
**Age group** (years)		
<5	15.7% (31/198)	8.2% (5/61)
5–14	36.4% (72/198)	42.6% (26/61)
15–29	27.3% (54/198)	29.5% (18/61)
30–44	12.1% (24/198)	11.5% (7/61)
45+	8.6% (17/198)	8.2% (5/61)
**Occupation**		
Child/student	59.2% (109/184)	60.9% (28/46)
Agricultural work	26.6% (49/184)	34.8% 16/46)
Non-agricultural work	6.0% (11/184)	0% (0/46)
Home/unemployed	8.1% (15/184)	4.3% (2/46)
**Location**		
West Darfur	41.4% (82/198)	36.7% (22/60)
Central Darfur	22.7% (45/198)	26.7% (16/60)
East Darfur	17.2% (34/198)	10.0% (6/60)
North Darfur	10.1% (20/198)	18.3% (11/60)
South Darfur	8.6% (17/198)	8.3% (5/60)
**Symptoms**		
Fever*	99.5% (197/198)	84.6% (55/65)
Any bleeding	96.9% (190/196)	95.7% (45/47)
Epistaxis	57.1% (112/196)	58.3% (28/48)
Gum	10.6% 20/189	14.0% (7/50)
Hematemesis	51.3% (97/189)	61.5% (32/52)
Bloody diarrhoea*	10.1% (19/188)	23.4% (11/47)
Joint pain	39.1% (75/192)	53.9% (28/52)
Loss of consciousness	7.4% (14/189)	8.5% (4/47)
Loss of appetite	29.6% (51/192)	40.0% (20/50)
Headache	49.5% (96/194)	59.3% (32/54)
Convulsions	12.2% (23/189)	8.3% (4/48)
**Exposure**		
Contact with animals	14.8% (8/54)	No data
**Case Fatality**	8.2% (16/196)	6.4% (3/47)
**Onset to admission** median (IQR) (n)	2 (1–3) days (181/198)	2 (1–3) days (41/65)
**Onset to sampling** median (IQR) (n)	4 (2–6) days (189/198)	4 (2–7) days (46/65)

Missing data is indicated in the table by the fluctuating denominator (n). In the onset to admission statistic, 15/17 missing cases among the non-transferred and 5/24 of the cases transferred to RIPL were not admitted.

* Significant difference between the two groups p = > 0.05

### Representativeness of tested samples

Sixty-five (24.7%) of the samples met the criteria for transfer for testing at RIPL. These were largely representative of the total cases sampled. There were no significant demographic differences, nor differences in the time from symptom onset to sample-taking, in the assays used by NPHL on the sample during the outbreak, or in their results or case outcome (Table 2). However, the transferred samples were associated with significantly less fever (p <0.001) and more bloody diarrhoea (p = 0.02) than those not transferred. It must be noted that, although a case definition was established during the outbreak, not all notified cases corresponded to it when analysed.

Case fatality was similar in both transferred and non-transferred samples (6.4%, 3/47 and 8.2%, 16/196 respectively, p = 1.0), but outcome data were missing in 18/65 cases in transferred group (27.7%) and 2/198 in non-transferred samples (1.01%).

Only 8 cases overall–none of which were among the transferred samples–reported any animal contact, but over 75% of data for this variable were missing. No other exposure variables were routinely reported, though early investigators reported seeing no epidemiological patterns, such as family clusters or healthcare worker infections, that might indicate person-to-person transmission.

### Laboratory results

Of the 15 tests performed on the 65 samples transferred to RIPL (Fig 3 & S1 Text), only the RT-PCR and ELISA for CCHFV produced positive results. Seven (11%) samples were positive on both RT-PCR and IgG ELISA, including 2 samples which had previously tested negative for CCHFV at NPHL. Cycle thresholds ranged from 23.90 to 36.02. Six of the 7 were also ELISA IgM positive. An additional 4 samples were RT-PCR and IgM-negative but positive for CCHFV IgG antibodies. All other samples tested negative on all assays.

**Fig 4:**
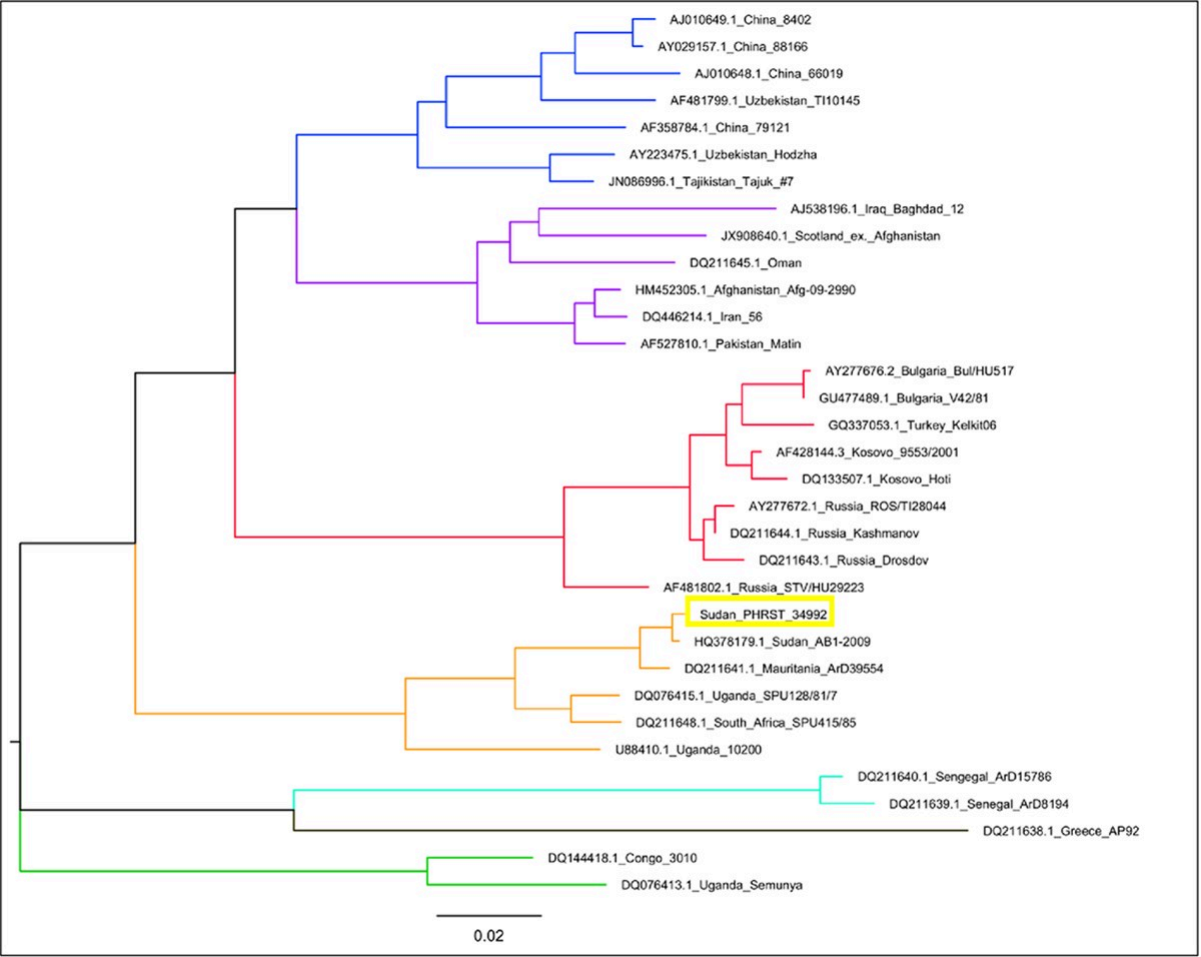
Maximum-likelihood phylogenetic tree showing relationship distances obtained by comparing CCHFV S Segment sequences. Previously defined major groups are indicated by branch colour (Asia 1,2 –Purple, Blue; Africa 1,2,3 –Green, Turquoise, Amber; Europe 1,2- Red, Brown).

No significant results were initially obtained through the metagenomic sequencing of either the CCHFV positive or negative samples due to inadequate RNA quality after storage and PCR work. However, when fresh nucleic acid extracts were made from primary samples for the CCHFV positive samples, sequencing on an Illumina MiSeq was successful, and confirmed that sequence reads identifiable as CCHFV were present in all 7 samples. Genome coverage varied widely between the samples (0–99.5% for the S segment, 0–96.5% for the M and 5.5–98.5% for the L, at 5x minimum depth) but produced adequate information to characterise the virus. Phylogenetic analysis of the near-complete S-segment of the sample with the highest quality information (S segment: 99.5%; M segment: 96.5%; L segment: 98.5%), dated 18 October in Table 3: male, 5 years, Ct 23.9), placed it within the Africa 3 S Segment clade, and showed close homology to the 2009 Sudan strain (HQ378179.1). (Genbank accession nos. S Segment: MK442893; M Segment: MK442894; L Segment: MK442895). See Fig 4 and S1 Text for further details.

**Table 3:** Characteristics of CCHFV positive cases identified by RIPL.

State	Date of onset (2015)	* Sex	Age	Occup.	Outcome	PCR cycle threshold		IgM +		IgG +	Fever	Any bleeding	Epistaxis	Bleeding gums	Blood in vomit	Bloody diarrhoea	* Bleeding rectum	Joint pain	Unconscious	Loss of appetite	Headache	Convulsions
N.Darfur	18 Oct	M	5	Child** ^¤^	Died	23.9	Neg		Pos		**Y**	**Y**	N	**Y**	N	N	.	N	**Y**	N	**Y**	**Y**
	10 Oct	M	30	Farmer**	Survived	32.0	Pos		Pos		**Y**	**Y**	**Y**	N	**Y**	**Y**	.	N	N	N	N	N
S. Darfur	Unkn	M	27	Unkn	Unkn.	29.4	Pos		Pos		.	.	.	.	.	.	.	.	.	.	.	.
*E.Darfur	30 Oct	M	28	Farmer	Alive	28.6	Pos		Pos		**Y**	**Y**	**Y**	N	N	N	N	**Y**	N	N	N	N
	Unkn	M	20	Unkn	Unkn	33.3	Pos		Pos		**Y**	.	.	**Y**	**Y**	.	.	.	.	**Y**	.	.
	11 Nov	M	25	Farmer ^¤^	Alive	36.0	Pos		Pos		**Y**	**Y**	N	N	N	**Y**	N	N	N	N	N	N
C.Darfur	13 Sep	M	21	Farmer ^¤^	Alive	29.3	Pos		Pos		**Y**	**Y**	N	N	**Y**	N	N	N	N	N	N	N

*Significant difference between CCHFV positive and negative samples

**Tested negative for CCHFV at NPHL during the outbreak

^¤^ Reported malaria positive

signifies missing data

Serological assays performed by NPHL during the outbreak on some of the study samples identified evidence of dengue antibodies in 23.2% (13 of 56 tested samples), chikungunya virus (1/65), hepatitis E virus (1/7) and West Nile virus (1/1). None of these infections were detected in RIPL testing. The only potential for co-infection seen was malaria. Clinical records at the time reported that 62.9% of the samples transferred to the UK were malaria-positive, including three of the cases subsequently shown to be positive for CCHF. Malaria testing was not repeated in RIPL.

### Characteristics of CCHFV positive cases

All 7 CCHFV RT-PCR positive/IgM positive cases were males aged 21 to 30 years, except for a 5-year-old boy who was also the only reported death in the group (CFR 20.0% of the 5 cases with reported outcome) (Table 3). The fatality was one of three CCHFV cases reported to be coinfected with malaria. Four of the positive cases were farmers and 2 were of unknown occupation. Three came from East Darfur, 2 from North Darfur and 1 each from South and Central Darfur. Of the 4 cases who were only CCHFV IgG positive, 1 came from each of North, South and East Darfur and the origin of the 4^th^ was unknown. Two of the 4 worked in animal husbandry.

Acute CCHFV positive cases were similar to CCHFV negative cases in most characteristics and symptoms, including the time from symptom onset to admission to a health facility and to sample-taking.

The only significant associations with acute CCHFV positivity were male sex (p 0.02) and origin in the state of East Darfur (p 0.002). The association with location was reinforced when RIPL-identified CCHFV positive cases were added to those identified during the outbreak by NPHL: when combined, 23% of all samples originating from East Darfur during the outbreak (9/40) were CCHFV positive and occurred during a 9-week period (September 28, 2015—January 12, 2016). NPHL-identified CCHFV positives originated from 2 main localities in East Darfur: RIPL-identified positives occurred in similar areas. Unfortunately, epidemiological data are not complete enough to investigate possible transmission links. CCHFV positive cases were less likely to report rectal bleeding than CCHFV negative cases (p 0.04) but half the CCHFV positive cases had missing data for this symptom.

## Discussion

Comprehensive diagnostic evaluation of this set of historical samples has demonstrated that CCHFV was one important cause, but not the only aetiology, of the large outbreak of undifferentiated febrile illness with haemorrhagic symptoms in Darfur in the 2015–2016. This fact that CCHFV was not the main cause is reinforced by the absence of CCHFV-positive samples from West Darfur, the state reporting the majority of cases in the outbreak. The substantial proportion of CCHFV-positive samples from East Darfur might suggest that the virus played a stronger role in the outbreak there, or that it simply represents background transmission in an endemic area. The 4 cases who were only CCHFV IgG positive most likely represent previous CCHFV exposure. It is also important to note that we found no evidence of DENV infection despite several previous outbreaks in the area having been attributed to this pathogen.

CCHFV-specific antibodies have been found in 19.1% of cattle in East Darfur and 21.3% of camels in Khartoum State, as well as in camels, sheep and goats exported from Sudan.[24,25] The first human cases of CCHFV in Sudan were confirmed during an outbreak of haemorrhagic fever among healthcare workers in Al Fulah Rural Hospital, Western Kordofan in 2008 with a CFR of 69.2%.[26] Since then, an average of 12 cases of human CCHFV have been confirmed by NPHL per year and multiple strains have been identified.[27] However, the persistent lack of laboratory capacity and supply at central level and complete absence of testing at district level mean that cases are likely to be under-reported.

The CFR of 20.0% among the CCHFV positive cases identified in this outbreak is at the lower end of the reported range for comparable resource-limited treatment settings. It is lower than might be expected given the severity of the reported illness[28,29], but similar to mortality in highly endemic regions such as Kazakhstan and Tajikistan.[30] One explanation may be that the severely ill and dead were less likely to be sampled, a possibility supported by the much higher mortality among outbreak cases reported but not sampled (24.1%). The only fatal case identified was in a 5year-old child with haemorrhage, and reportedly coinfected with malaria, who was admitted two days after onset of symptoms, who tested negative by PCR at the NPHL but subsequently positive by PCR and sequencing at RIPL and died 3 days after admission. While fatal outcome of CCHF in children is thought to be rare it has been reported from a range of settings.[31–33]

An explanation for the finding of positive CCHFV results in two samples previously tested ‘negative’ at NPHL could be that CT values were borderline, but unfortunately documentation at that time only reported results as negative or positive. It is also possible that the assays used (Altona real time PCR and Euroimmune ELISA) could have been at the limit of their validity as NPHL have had great difficulty in maintaining reagent supply and conditions during the embargo in Sudan and with frequent power cuts and equipment failure.

The importance of livestock to the Sudanese economy and the risks that CCHFV presents to healthcare workers and other patients[34], especially in resource-limited healthcare facilities, highlight the urgent need for improved surveillance and faster, more accessible CCHFV laboratory diagnosis. But while our comprehensive testing clearly highlights the risks and response requirements for CCHFV and has underlined some important negative results which excluded other HCIDs and VHFs, it has not solved the conundrum of what caused the majority of cases.

The absence of a diagnosis for the majority of these historical specimens may result from compromised sample integrity in transportation from the field to the NPHL, or during the storage period (18–24 months) between sample collection and final laboratory analysis. While the NPHL’s receipt of samples for almost 50% of reported cases is remarkable given the challenges in surveillance and outbreak response in these low-resourced and remote settings, the inherent delays and difficulties with cold chain take their toll on sample condition. It is also possible that delayed presentation to health facilities could have led to sampling later in the disease course when opportunity to identify pathogens by PCR is reduced. A further factor difficult to assess is the impact of genetic variation and primer and/or probe mismatches during the PCR testing which may have affected opportunity for positive findings. The extent of our laboratory work and our sample size was also limited by the relatively small amounts of stored sample available. This combined with the absence of convalescent samples precluded more detailed serological analysis.

Increasing the capacity to investigate samples on site through the development of innovative near-patient diagnostic platforms—a priority in the WHO R &D Blueprint for Action to Prevent Epidemics[35]—and/or improving storage and transfer of samples to the reference laboratory, will be key to rapid identification of pathogens in future outbreaks. Additional diagnostic capacity achieved by increasing the range of assays available in the NPHL, collecting a broader range of field samples (e.g. urine, naso-oropharangeal and stool samples as well as convalescent samples) and a developing a sequencing capability, will also increase diagnostic yield.

Our interpretation of the syndrome characteristics was limited by missing epidemiological data. The absence of full case investigation forms (CIF) for 19 of the 65 investigated samples restricted characterisation of symptoms, occupation and outcomes. Exposures and contact information were also rarely recorded–an important oversight for outbreak control–and similar or greater gaps in data were found in the information of the un-transferred cases. Using a more detailed CIF in future outbreaks, ideally in electronic format to speed interpretation, and reinforcing the importance of face-to-face interviews with patients, family and community will help the capture of important data. To assist investigation of future outbreaks, the UK Public Health Rapid Support Team/FMoH research collaboration has put in place an ethics-approved prospective study protocol and trained a study team comprising FMoH, NPHL and University staff ready to deploy.

In conclusion, Sudan is a large country at the crossroads of Africa and the Middle East that has experienced several outbreaks of HCIDs and regularly has outbreaks of zoonotic infections. We have shown the presence of CCHFV as an important cause of fever and haemorrhage in Darfur which has critical infection prevention and control as well as clinical implications for future care and response. To fulfil its global obligations under the International Health Regulations (2005), the FMoH needs to maintain and develop robust surveillance, response and laboratory capacity. Support in these areas, and for well-developed prospective fever studies, could dramatically improve understanding of circulating pathogens in Sudan and facilitate the rapid recognition and investigation of the next outbreak.

## Supporting information

S1 Text Supporting information: Contextual and Technical Appendix: detailed laboratory and metagenomic materials and methods. (DOCX)

S2 Text Strobe checklist. (DOCX)

## References

[1] Crump JA, Kirk MD. Estimating the Burden of Febrile Illnesses. PloS neglected tropical diseases. 2015 12;9(12):e0004040 10.1371/journal.pntd.0004040 26633014 PMC4668833

[2] Maze MJ, Bassat Q, Feasey NA, Mandomando I, Musicha P, Crump JA. The epidemiology of febrile illness in sub-Saharan Africa: implications for diagnosis and management. Clinical Microbiology and Infection. 2018;24(8):808–14 10.1016/j.cmi.2018.02.011 29454844 PMC6057815

[3] Shrestha P, Roberts T, Homsana A, Myat TO, Crump JA, Lubell Y, et al Febrile illness in Asia: gaps in epidemiology, diagnosis and management for informing health policy. Clinical Microbiology and Infection. 2018;24(8):815–26 10.1016/j.cmi.2018.03.028 29581051

[4] Undiagnosed febrile illness, fatal—Sudan: (SF) RFI. ProMed. 2012, Oct 23. http://www.promedmail.org, archive no. 20121023.1360013

[5] Undiagnosed disease—Sudan: (Darfur) RFI. ProMed. 2012, Dec 23. http://www.promedmail.org, archive no. 20121223.1465553

[6] Yellow fever—Africa (27): Sudan, conf. ProMed. 2013, Nov 28. http://www.promedmail.org, archive no. 20131128.2081032

[7] Viral hemorrhagic fever—Sudan: (Darfur) RFI. ProMed. 2013, Oct 16. http://www.promedmail.org, archive no. 20131016.2005262

[8] Undiagnosed hemorrhagic fever—Sudan (03): (SF) displaced persons. ProMed. 2014, Nov 1. http://www.promedmail.org, archive no. 20141101.2921031

[9] Undiagnosed hemorrhagic fever—Sudan (02): (West Kordofan) RFI. ProMed 2014, Oct 28. http://www.promedmail.org, archive no. 20141028.2907268

[10] Undiagnosed hemorrhagic fever—Sudan (03): (SF) displaced persons, RFI ProMed. 2014, Nov 1. http://www.promedmail.org, archive no. 20141101.2921031

[11] Undiagnosed hemorrhagic fever—Sudan (05): (South Kordofan) RFI. ProMed. 2014, Nov 11. http://www.promedmail.org, archive no. 20141111.2946812

[12] Undiagnosed hemorrhagic fever—Sudan (06): Gezira, RFI. ProMed. 2014, Nov 19. http://www.promedmail.org, archive no. 20141119.2972219

[13] Undiagnosed hemorrhagic fever—Sudan (03): (Darfur) RFI ProMed. 2015, Nov 2. http://www.promedmail.org, archive no. 20151102.3760440

[14] Undiagnosed hemorrhagic fever—Sudan (04): (North Dafur): RFI. ProMed. 2015, Nov 8. http://www.promedmail.org, archive no. 20151108.3775050

[15] Undiagnosed hemorrhagic illness—Sudan: (KA) RFI. ProMed. 2017, Nov 14. http://www.promedmail.org, archive no. 20171114.5444239

[16] Undiagnosed disease—Sudan (south) (04). ProMed. 2003 May 17. http://www.promedmail.org, archive no. 20030517.1224

[17] Undiagnosed illness—South Sudan: (Jonglei) fatal, RFI. ProMed. 2013, Oct 3. http://www.promedmail.org, archive no. 20131003.1981265

[18] Undiagnosed hemorrhagic illness—South Sudan ProMed. 2016, May 19. http://www.promedmail.org, archive no. 20160519.4233420

[19] World Health Organisation. Ebola haemorrhagic fever in Sudan, 1976. Report of a WHO/International Study Team. Bull World Health Organ. 1978;56(2):247–70 307455 PMC2395561

[20] World Health Organization. Ebola haemorrhagic fever in Zaire, 1976. Report of an International Commission. Bull World Health Organ. 1978;56(2):271–93 307456 PMC2395567

[21] Markoff L. Yellow fever outbreak in Sudan. The New England journal of medicine. 2013;368:689–91 10.1056/NEJMp1300772 23387798

[22] Sabahelzain MM, Hamamy H. The ethnic distribution of sickle cell disease in Sudan. The Pan African Medical Journal. 2014;18:13 10.11604/pamj.2014.18.13.3280 PMC4213521 25360197

[23] Kafetzopoulou LE, Efthymiadis K, Lewandowski K, Crook A, Carter D, Osborne J, et al Assessment of metagenomic Nanopore and Illumina sequencing for recovering whole genome sequences of chikungunya and dengue viruses directly from clinical samples. Eurosurveillance. 2018;23(50):1800228 10.2807/1560-7917.ES.2018.23.50.1800228 PMC6299504 30563591

[24] Gasemallah AMI. Serological Survey of Crimean Congo Hemorrhagic Fever and Associated Risk Factors among Cattle in East Darfur State, Sudan. University of Khartoum, Sudan 2015.

[25] Suliman HM, Adam IA, Saeed SI, Abdelaziz SA, Haroun EM, Aradaib IE. Crimean Congo hemorrhagic fever among the one-humped camel (Camelus dromedaries) in Central Sudan. Virology Journal. 2017 8 03;14(1):147 10.1186/s12985-017-0816-3 28774342 PMC5543554

[26] Aradaib IE, Erickson BR, Mustafa ME, Khristova ML, Saeed NS, Elageb RM, et al Nosocomial Outbreak of Crimean-Congo Hemorrhagic Fever, Sudan. Emerging Infectious Diseases. 2010;16(5):837–9 10.3201/eid1605.091815 20409377 PMC2954009

[27] Aradaib IE, Erickson BR, Karsany MS, Khristova ML, Elageb RM, Mohamed MEH, et al Multiple Crimean-Congo Hemorrhagic Fever Virus Strains Are Associated with Disease Outbreaks in Sudan, 2008–2009. PloS neglected tropical diseases. 2011;5(5):e1159 10.1371/journal.pntd.0001159 21655310 PMC3104971

[28] Al-Abri SS, Abaidani IA, Fazlalipour M, Mostafavi E, Leblebicioglu H, Pshenichnaya N, et al Current status of Crimean-Congo haemorrhagic fever in the World Health Organization Eastern Mediterranean Region: issues, challenges, and future directions. International Journal of Infectious Diseases. 2017 2017/05/01/;58:82–9 10.1016/j.ijid.2017.02.018 28259724 PMC7110796

[29] Leblebicioglu H, Sunbul M, Guner R, Bodur H, Bulut C, Duygu F, et al Healthcare-associated Crimean-Congo haemorrhagic fever in Turkey, 2002–2014: a multicentre retrospective cross-sectional study. Clin Microbiol Infect. 2016;22(4):387.e1–.e4 26806137 10.1016/j.cmi.2015.11.024 PMC5023843

[30] Nurmakhanov T, Sansyzbaev Y, Atshabar B, Deryabin P, Kazakov S, Zholshorinov A, et al Crimean-Congo haemorrhagic fever virus in Kazakhstan (1948–2013). Int J Infect Dis. 2015 9;38:19–23 10.1016/j.ijid.2015.07.007 26183415

[31] Ahmeti S, Raka L. Crimean-Congo Haemorrhagic Fever in Kosova: a fatal case report. Virology Journal. 2006 10 12;3(1):85 17034648 10.1186/1743-422X-3-85 PMC1624825

[32] Belet N, Top A, Terzi O, Arslan HN, Baysal K, Sensoy G. Evaluation of children with Crimean-Congo hemorrhagic fever in the central Blacksea region. Pediatr Infect Dis J. 2014 8;33(8):e194–7 10.1097/INF.0000000000000281 24463803

[33] Aslani D, Salehi-Vaziri M, Baniasadi V, Jalali T, Azad-Manjiri S, Mohammadi T, et al Crimean-Congo hemorrhagic fever among children in Iran. Archives of Virology. 2017 3 01;162(3):721–5 10.1007/s00705-016-3162-7 27878461

[34] Fletcher TE, Gulzhan A, Ahmeti S, Al-Abri SS, Asik Z, Atilla A, et al Infection prevention and control practice for Crimean-Congo hemorrhagic fever—A multi-center cross-sectional survey in Eurasia. PLOS ONE. 2017;12(9):e0182315 10.1371/journal.pone.0182315 28886039 PMC5590734

[35] World Health Organisation. An R&D Blueprint for Action to Prevent Epidemics: Plan of Action https://www.who.int/blueprint/en/, accessed 13 May 2019.

