# Supplementary material for: Detection of Crimean-Congo Haemorrhagic Fever cases in a severe undifferentiated febrile illness outbreak in the Federal Republic of Sudan: A retrospective epidemiological and diagnostic cohort study
Source: PLoS Negl Trop Dis. 2019 Jul 10;13(7):e0007571. doi: 10.1371/journal.pntd.0007571 (PMC6645580; doi:10.1371/journal.pntd.0007571)
Supplement: S1 Text — (DOCX) [file pntd.0007571.s001.docx]

**Detection of Crimean-Congo Haemorrhagic Fever cases in a severe undifferentiated febrile illness outbreak in the Federal Republic of Sudan: a retrospective epidemiological and diagnostic cohort study**

**S1 Supporting Information**

**Contents**

1. Table 1. Viral haemorrhagic fever outbreaks reported by the Federal Ministry of Health of Sudan, 1976 – 2015 (excluding outbreaks of ‘unknown’ cause reported in Table 1 of the main text
2. Technical Appendix:

Detailed laboratory and metagenomic materials and methods

Table 2: Molecular assays used in investigation of Sudanese legacy samples

Table 3: Genome coverage at 1X and 5X depth for each CCHFV positive sample.

**Table A: Confirmed and suspected viral haemorrhagic fever outbreaks reported by the Federal Ministry of Health of Sudan, 1976 – 2018 (excluding outbreaks of ‘unknown’ cause reported in Table 1 of the main text)**

| **No of Death (%)** | **No. of Reported Cases** | **Suspected or confirmed infection** | **State affected** | **Year** |
| --- | --- | --- | --- | --- |
| 151 (53.1) | 284 | Ebola | West Equatoria | 1976 |
| 22 (66.0) | 33 | Ebola | East Equatoria | 1979 |
| - | Sporadic | Dengue fever | Red Sea, Kassala | Before 2003 |
| 4 (31.0) | 13 | Ebola | Bahr El Gabal | 2004 |
| 163 (26.9) | 605 | Yellow fever | South Kordofan | 2005 |
| 214 (31.8) | 673 | RVF | White Nile, Gazeera | 2007 |
| 49 (51.0 | 96 | CCHF | South Kordofan | 2008 |
| 25 (5.6) | 449 | Dengue fever | Red Sea, Kassala | 2008 |
| 12 (0.4) | 3300 | Dengue fever | Red Sea, Kassala | 2010 |
| - | Sporadic | West Nile fever | South Kordofan | 2011 |
| 5 (41.7) | 12 | CCHF | Eastern Darfur | 2014 |
| Unkn | 201 | Dengue fever | Kassala | 2017 |
| 1 (50%) | 2 | CCHF | Blue Nile | 2018 |
| * | 19,897 | Chikungunya | Kassala City | 2018 |

* Reports have varied from 0 to 7 deaths (CFR - 0.04%)

**B. Technical Appendix: Detailed laboratory and metagenomic materials and methods**

***RNA extraction***

Plasma samples were thawed and, if haemolysed, were clarified by centrifugation in a table-top centrifuge at 3000 × g for 10 min. 80 µL plasma was inactivated in 320 µL AVL buffer (Proprietary Guanidinium HCl buffer, Qiagen Ltd, Manchester, UK) for 10 mins at room temperature. Nucleic acid was extracted in the presence of 3 µg carrier RNA and in-house exogenous internal control template [intact MS2 bacteriophage(*1, 2*) and *E.coli*-green fluorescent protein (*E.coli* BLR-GFP *(3, 4)*], using the EZ1 Virus Mini Kit v2.0 on an automated EZ1 Advanced XL platform (Qiagen). Purified nucleic acid was eluted in 120 µL AVE buffer (Proprietary Tris-EDTA buffer, Qiagen) and used immediately or stored at -20^o^C.

***PCR analysis***

All 65 extracts were analysed in parallel. PCR tests were prioritized and performed in the following sequence: (1) CCHFV and DENV-1-3 duplexed with MS2, (2) DENV-4, YFV, RVFV and rickettsiae; (3) Ebolaviruses and Marburgviruses; (4) *Leptospira* spp; (5) *Borrelia* spp. (6) Bunyamwera viruses (BUNV) and Alkhurma virus (ALKV) and (7) Old World arenaviruses. Brief details of the design of each assay are given below. Primer and probe sequences, together with details of the individual optimized PCR assay conditions, are provided in Table 1. With the exception of leptospirosis, all reactions used 5 µL of template in a final 20 µL reaction.

Intact MS2 bacteriophage(*2*) and *E. coli* BLR-GFP(*3, 4*) were included in all extractions as exogenous internal controls. MS2 RNA detection was duplexed with DENV-1-3; GFP template was detected as part of the leptospirosis LipL32 assay. Each PCR run included extract negative samples (exogenous control only), non-template (PCR-grade water) negative controls and relevant positive amplification controls, typically in-house designed synthetic plasmids (synthesised by GeneArt, InVitrogen).Intact MS2 bacteriophage *(2)* and *E.coli* BLR-GFP *(3, 4)* were included in all extractions as exogenous internal controls. MS2 RNA detection was duplexed with DENV1-3; GFP template was detected as part of the leptospirosis LipL32 assay *(3)*. Each PCR run included extract negative samples (exogenous control only), non-template (PCR-grade water) negative controls and relevant positive amplification controls, typically in-house designed synthetic plasmids (synthesised by GeneArt, InVitrogen).

Detection of CCHFV RNA was performed using a real-time TaqMan reverse transcription (RT)-PCR assay designed to detect the highly conserved panhandle loop formation of the CCHFV S segment *(5)* which detects a broad geographic range of CCHFV RNA.

Real-time PCR detection of DENV-1-3 (duplexed with MS2 exogenous control), DENV-4 and YFV RNA and *Rickettsia* spp. DNA used published assays (*6, 7*); for RVFV an unpublished assay was used. All reactions shared the same cycling conditions; these are summarized in table 1.

Two assays to detect pan-Ebolavirus and pan-Marburgvirus RNA were based on the published assay described by Panning *et al*. *(1).* Degenerate base pairing was used to redesign single forward and reverse PCR primers and a minor groove binder hydrolysis probe (Taqman-MGB) for each virus genus (Table 1).

In-house real time RT-PCR assays were used for detection of ALKV and BUNV RNA (Table 1); the latter was designed to pick up all strains of BUNV known to cause human infection, including reassortants such as Ngari virus.

For viral and rickettsial PCRs, results in the relevant fluorescence channel were analysed and typically the following interpretation was applied: samples with no amplification or with a Cycle threshold (Ct) value >40 and no sample inhibition as verified by the MS2 internal control (performed in parallel or multiplexed), were interpreted as negative. Samples with acceptable amplification curves and Ct ≤ 35 were interpreted as positive. Samples with late amplification curves and (Ct > 35 and <40) were re-extracted and re-tested in duplicate, and if still detectable were assigned an equivocal interpretation.

For detection of *Leptospira* spp. DNA, an in-house multiplex real-time PCR assay was used *(3)* comprising 2 PCRs. First, a duplex PCR targeting the *LipL*32 gene that encodes an outer membrane lipoprotein reported to be strongly associated with the pathogenic phenotype (1) and the GFP template added as an internal process control during nucleic acid extraction. (2) Second, a triplex assay targeting a well conserved region within the 16S rRNA gene (*rrn*) of leptospires using 3 differentially labelled probes to detect genomic variants with differing pathogenicity. PCRs were performed with 5 $\mu$L nucleic acid in a 25 µL final reaction mix. Samples with Ct > 45 and no inhibition in the exogenous GFP control (Cy5 channel in LipL32 assay) were interpreted as negative. Clinical samples with Ct values between 14 and 45 with acceptable amplification curves were interpreted as positive, with the lowest probe Ct used to assign the genomic variant. The 16s rRNA assay is more sensitive than the Lipl32 assay.

Detection of *Borrelia* spp. DNA used a real-time PCR assay which targets a region of the 16S rRNA *Borrelia* gene which is conserved across all known pathogenic species within the *Borrelia* genus *(8)* It is capable of detecting all members of the *Borrelia burgdorferi* sensu lato genospecies complex, *B. miyamotoi,* and species that cause tick-borne relapsing fever (e.g. *B. hermsii, B. duttonii*). Similar interpretation criteria were applied as for the viral PCRs.

RT-PCR for Old World arenaviruses used a published block-based assay *(9)* with slight modifications to oligonucleotide sequences and reaction conditions *(10)* (Table 1). This has been shown to detect RNA from Lassa virus, Lujo virus, Lymphocytic choriomeningitis virus and Mopeia virus. PCR products were visualized on a 1 % (w/v) Tris-acetate EDTA gel supplemented with 1 X SYBR Safe under UV light.

**Table B: Molecular assays used in investigation of Sudanese legacy samples**

| **Pathogen** | **Ref** | **Primer/probe** | **Name** | **Sequence (5’ – 3’)** | **Genomic target** | **Final conc.**  **(nM)** | **Thermal cycling conditions** |
| --- | --- | --- | --- | --- | --- | --- | --- |
| CCHFV | *(5)* | F | CCHF S122F | CCT TTT TGA ACT CTT CAA ACC | S segment | 900 | SuperScript III Platinum One-Step qRT-PCR Kit  Roche Light Cycler 2.0  1x 50^o^C for 10 min  1x 95^o^C for 2 min  45x (95^o^C for 10 sec; 60^o^C for 40 sec)  1x 40^o^C for 30 sec |
|  |  | R | CCHF S1R | TCT CAA AGA AAC ACG TGC C |  | 900 |  |
|  |  | P | CCHF Probe | FAM-ACT CAA GGK AAC ACT GTG GGC GTA AG-BHQ1-NFQ |  | 625 |  |
| DENV1-3 | *(6)* | F | DEN Dros F | GGA TAG ACC AGA GAT CCT GCT GT | 3′ noncoding region | 300 | SuperScript III Platinum One-Step qRT-PCR Kit  Roche Light Cycler 480  1x 45^o^C for 10 min  1x 95^o^C for 5 min  45x (95^o^C for 5 sec; 57^o^C for 35 sec)  1x 40^o^C for 30 sec |
|  |  | R | DEN Dros R1 | CAT TCC ATT TTC TGG CGT TC |  | 900 |  |
|  |  | P | Den Dros P | FAM-CAG CAT CAT TCC AGG CAC AG-BHQ1 |  | 500 |  |
| MS2 | *(2)* | F | MS2 F1 | TGG CAC TAC CCC TCT CCG TAT TCA CG | MS2 genome (nt 289-387) | 40 |  |
|  |  | R | MS2 Rev | GTA CGG GCG ACC CCA CGA TGA C |  | 40 |  |
|  |  | P | MS2 taq Cy5 | Cy5 CAC ATC GAT AGA TCA AGG TGC CTA CAA GC BBQ1 |  | 80 |  |
| DENV4 | *(6)* | F | DEN Dros F | GGA TAG ACC AGA GAT CCT GCT GT | 3′ noncoding region | 50 |  |
|  |  | R | DEN Dros R2 | CAA TCC ATC TTG CGG CGC TC |  | 450 |  |
|  |  | P | Den Dros P | FAM-CAG CAT CAT TCC AGG CAC AG-BHQ1 |  | 500 |  |
| YFV | *(6)* | F | TM YF FWD | AAT CGA GTT GCT AGG CAA TAA ACA C | 5′ noncoding region | 250 |  |
|  |  | R | TM YF REV | TCC CTG AGC TTT ACG ACC AGA |  | 250 |  |
|  |  | P | TM YF PRB | FAM-ATC GTT CGT TGA GCG ATT AGC AG- BHQ1 |  | 200 |  |
| RVFV | -* | F | RVFNF | GGA TAG GCC GTC CAT GGT AGT | N gene | 900 |  |
|  |  | R | RVFNR | GGA CAT GCC AGG CGY TGG T |  | 900 |  |
|  |  | P | RVFNP | FAM-CCA GTG ACA GGA AGC CAC TCA CTC AAG A-BHQ1 |  | 625 |  |
| *Rickettsia* spp. | *(7)* | F | CS-F | TCG CAA ATG TTC ACG GTA CTT T | *GltA* gene | 900 |  |
|  |  | R | CS-R | TCG TGC ATT TCT TTC CAT TGT G- |  | 450 |  |
|  |  | P | CS-P | FAM-TGC AAT AGC AAG AAC CGT AGG CTG GAT G-BHQ1 |  | 600 |  |

| Ebolaviruses | *(1)* | F | FiloA_Ebola | AAG CMT TTC CHA GCA AYA TGA TGG T | L gene | 900 | TaqMan Fast Virus 1-step master mix  ABI ViiA 7 PCR system  1x 50^o^C for5 min  1x 95^o^C for 20 sec  40x (95^o^C for 3 sec, 60^o^C for 30 sec) |
| --- | --- | --- | --- | --- | --- | --- | --- |
|  |  | R | FiloB_Ebola | ATG HGG TGG ATT ATA ATA ATC ACT DAC ATG |  | 900 |  |
|  |  | P | FAMEBO_DEGEN | FAM-CCR AAA TCA TCA CTB GTR TGG TGC CA-MGB |  | 250 |  |
| Marburgviruses | *(1)* | F | FiloA_Marburg | AAG CAT TCC CKA GTA ACA TGA TGG T | L gene | 900 |  |
|  |  | R | FiloB_Marburg | ATG WGG WGG GCT ATA AAA ATC ACT GAC ATG |  | 900 |  |
|  |  | P | FAMMBG | FAM-CCT ATG CTT GCT GAA TTG TGG TGC CA-MGB |  | 250 |  |
| Leptospirosis | *(3)* | F | LeptoF1 | GAT CGG TAR CCG GCC T | *rrn* gene (16S rRNA) | 400 | Takyon Low Rox Probe MasterMix dTTP Blue (Eurogentec, Belgium)  ABI ViiA 7 PCR system  1x 95^o^C for 5 min  50x (95^o^C for 3 sec, 60^o^C for 30 sec, 72^o^C for 10 sec) |
|  |  | F | LeptoF2 | CGA TCA GTA RCC GGC CT |  | 400 |  |
|  |  | R | LeptoR | CCC ATT GAG CAA GAT TCT TAA |  | 400 |  |
|  |  | P | Pathprobe | FAM-AGA GGG TGT TCG GCC ACA ATG-BHQ1 |  | 100 |  |
|  |  | P | Interprobe | JOE-AGA GGG TGT CCG GCC ACA AT-BHQ1 |  | 100 |  |
|  |  | P | Enviroprobe | CY5-AGA GGG TGA ACG GCC ACA ATG-BHQ2 |  | 100 |  |
| Leptospirosis | *(3)* | F | LipL32AF | GGA TCT GTG ATC AAC TAT TAC GGA TA | LipL32 gene | 400 |  |
|  |  | R | LipL32Arv2 | CTC CCA TTT CAG CGA TTA C |  | 400 |  |
|  |  | P | LipLprobe | FAM-TAA AGC CAG GAC AAG CGC CG-BHQ1 |  | 100 |  |
| GFP | *(3, 4)* | F | GFPF | CCT GTC CTT TTA CCA GAC AAC CA | GFP control target | 400 |  |
|  |  | R | GFPR | GGT CTC TCT TTT CGT TGG GAT C |  | 400 |  |
|  |  | P | GFPprobe | CY5-TAC CTG TCC ACA CAA TCT GCC CTT TCG-BHQ2 |  | 100 |  |
| BUNV | -* |  | BUN F | CCA GCA GTA CTT TTG ACC CAG AG | S segment | 500 | SuperScript III Platinum One-Step qRT-PCR  ABI 7500 Fast PCR system  1x 50^o^C for 5 min  1x 95^o^C for 20 s  45x (95^o^C for 3 s and 60^o^C for 30 s) |
|  |  |  | BUN R | CTT CCA GCC CCC AAG GTT AAG |  | 250 |  |
|  |  |  | BUN Pb | Cy5-AAT YTC KCG TCC TTT AAT GTA GAA GAT TC-BHQ3 |  | 250 |  |
| ALKV | -* |  | ALK RT F 1599 | GGT GCA CCG AGA CTG GTT | E gene | 500 |  |
|  |  |  | ALK RT R 1675 | GGT CAG CGT GGT TCC A |  | 500 |  |
|  |  |  | ALK RT P 1621 | FAM-GAC CTC TCC TTG CCA TGG CGA CA-BHQ1 |  | 500 |  |

| Old World arenaviruses | *(9, 10)* | F | Pan OW arenavirus F | AGA ATY AGT GAA AGG GAR AGY AAY TC | L segment | 500 | SuperScript III Platinum One-Step qRT-PCR Kit  Roche LightCycler 480 Instrument II  1x 50^o^C for 15 min  1x 95^o^C for 2 min  45x (95^o^C for 15 s, 50^o^C for 30 s, 68 ^o^C for 30 s) |
| --- | --- | --- | --- | --- | --- | --- | --- |
|  |  | R | Pan OW arenavirus R | CAC ATC ATT GGT CCC CAT TTA CTR TGA TC |  | 500 |  |
| *Borrelia* spp | *(8)* | F | Bor16S3F | AGC CTT TAA AGC TTC GCT TGT AG | 16S rRNA | 900 | TaqMan Fast Universal PCR master mix  ABI ViiA 7 with Fast 96-Well  1x 95^o^C for 20 sec  40x (95^o^C for 3 sec, 60^o^C for 30 sec) |
|  |  | R | Bor16S3R | GCC TCC CGT AGG AGT CTG G |  | 900 |  |
|  |  | P | Bor16S3P | FAM-CCG GCC TGA GAG GGT GAACGG-BHQ1 |  | 250 |  |

*Unpublished

Abbreviations: CCHFV: Crimean-Congo haemorrhagic fever virus; DENV: Dengue virus; MS2: MS2 bacteriophage; YFV: Yellow fever virus; RVF: Rift Valley fever virus; GFP: Green fluorescent protein; BUNV: Bunyamwera virus; ALKV: Alkhurma virus.

**CCHF ELISA**

Plasma samples in which CCHFV RNA was detected were immediately transferred to high containment (Containment Level - CL 4). Samples were heat-inactivated at 56°C for 30 minutes (with samples mixed midway), conditions shown to fully inactivate CCHFV.*(11)*. VectoCrimea-CHF ELISA kits (Vector-Best, Novosibirsk, Russia) were used for detection of CCHFV-specific IgG and IgM following the manufacturers’ instructions. Absorbances were read at an optical density of 450nm (OD450_nm_) on a microplate reader (SpectraMax M3, Molecular Devices, UK) and analysed using SoftMax Pro (version 7.0, Molecular Devices, UK). The cut-off value was calculated as the average OD_450nm_ of the negative controls supplied with the kit + 0.2. Samples were considered to be positive if OD_450nm_ of the sample was greater than or equal to this cut-off value.

**Genome Sequencing and phylogenetic analysis**

Sequencing methods and analysis performed are as described in detail by Kafetzopoulou et al.(12) Genome coverage achieved for each of the positive samples is outlined in Table 2 of this appendix. Phylogenetic analysis of the near-complete S segment obtained from sample 34992 along with representative genomes from the major CCHF lineages and Sudan is displayed in Figure 1. The evolutionary history was inferred using the Maximum Likelihood method and Tamura-Nei model in MEGA 6.06.(13) The CCHF S, M and L segment sequences obtained from this sample are available in Genbank (Accession Nos.: S Segment: MK442893; M Segment: MK442894; L Segment: MK442895.

**Table C: Genome coverage at 1X and 5X depth for each CCHFV positive sample.**

| **Sample** | **S Segment** | | **M Segment** | | **L Segment** | |
| --- | --- | --- | --- | --- | --- | --- |
|  | **1X Coverage (%)** | **5X Coverage (%)** | **1X Coverage (%)** | **5X Coverage (%)** | **1X Coverage (%)** | **5X  Coverage (%)** |
| 34981 | 39 | 17.8 | 3.6 | 0.8 | 20.7 | 12.6 |
| 34986 | 10.7 | 6.2 | 0 | 0 | 6.6 | 6.5 |
| 34992 | 99.8 | 99.5 | 98.4 | 96.5 | 99.9 | 98.5 |
| 35020 | 45.7 | 38.9 | 23 | 11.9 | 15.3 | 11.6 |
| 35026 | 0 | 0 | 0 | 0 | 7.6 | 5.5 |
| 35028 | 0 | 0 | 0 | 0 | 10.1 | 6.9 |
| 35037 | 72.1 | 63.3 | 35.3 | 28 | 37.5 | 33.4 |

Reads were mapped to a 2009 Sudanese CCHFV sequence as reference for small, medium and large segments (HQ378179.1, HQ378187.1 and HQ378183.1 respectively).


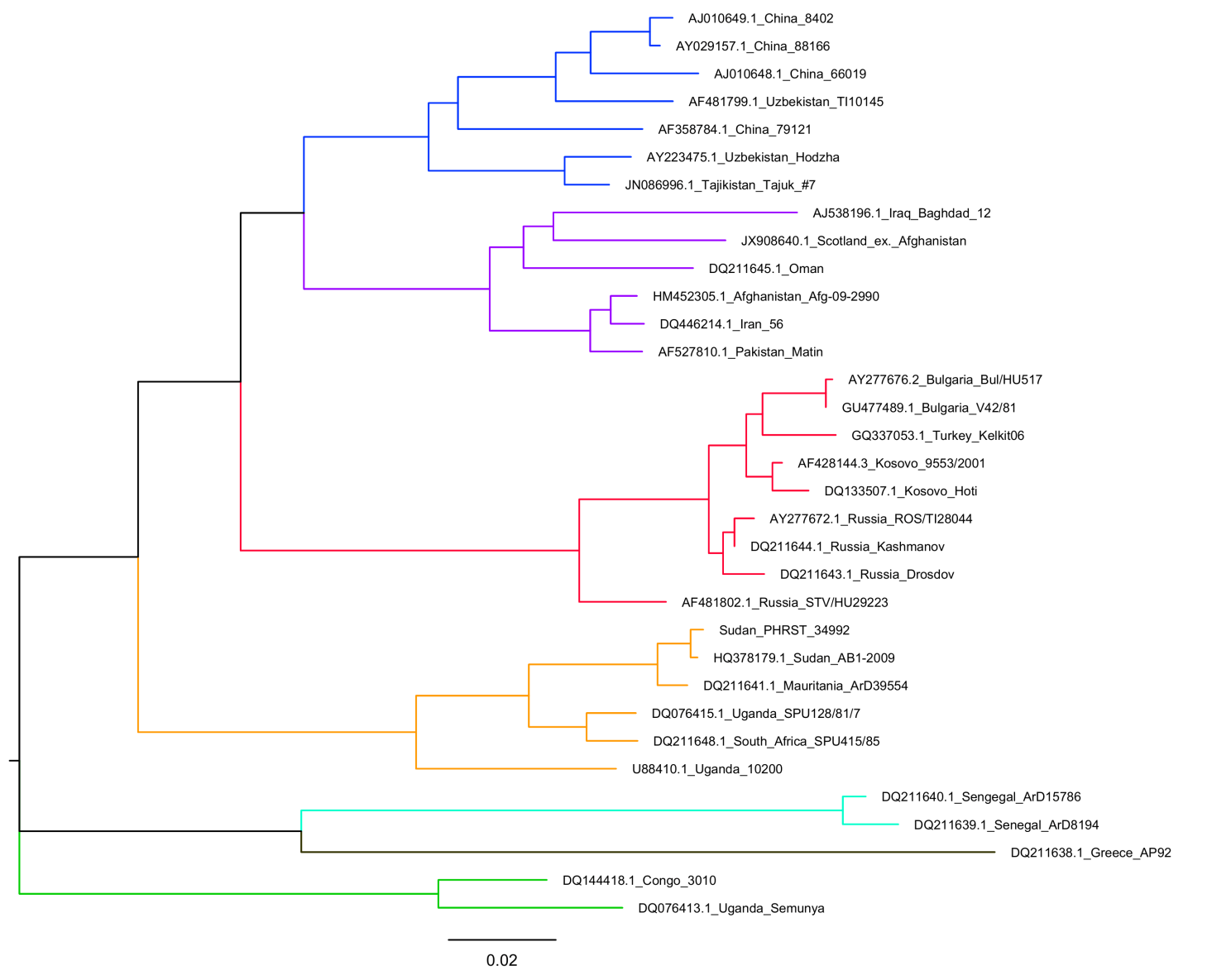


**References**

1. Panning M, Laue T, Olschlager S, Eickmann M, Becker S, Raith S, et al. Diagnostic reverse-transcription polymerase chain reaction kit for filoviruses based on the strain collections of all European biosafety level 4 laboratories. J Infect Dis. 2007;196 Suppl 2:S199-204.

2. Rolfe KJ, Parmar S, Mururi D, Wreghitt TG, Jalal H, Zhang H, et al. An internally controlled, one-step, real-time RT-PCR assay for norovirus detection and genogrouping. J Clin Virol. 2007;39(4):318-21.

3. Woods K, Nic-Fhogartaigh C, Arnold C, Boutthasavong L, Phuklia W, Lim C, et al. A comparison of two molecular methods for diagnosing leptospirosis from three different sample types in patients presenting with fever in Laos. Clin Microbiol Infect. 2018;24(9):1017 e1- e7.

4. Murphy NM, McLauchlin J, Ohai C, Grant KA. Construction and evaluation of a microbiological positive process internal control for PCR-based examination of food samples for Listeria monocytogenes and Salmonella enterica. Int J Food Microbiol. 2007;120(1-2):110-9.

5. Atkinson B, Chamberlain J, Logue CH, Cook N, Bruce C, Dowall SD, et al. Development of a real-time RT-PCR assay for the detection of Crimean-Congo hemorrhagic fever virus. Vector Borne Zoonotic Dis. 2012;12(9):786-93.

6. Drosten C, Gottig S, Schilling S, Asper M, Panning M, Schmitz H, et al. Rapid detection and quantification of RNA of Ebola and Marburg viruses, Lassa virus, Crimean-Congo hemorrhagic fever virus, Rift Valley fever virus, dengue virus, and yellow fever virus by real-time reverse transcription-PCR. J Clin Microbiol. 2002;40(7):2323-30.

7. Stenos J, Graves SR, Unsworth NB. A highly sensitive and specific real-time PCR assay for the detection of spotted fever and typhus group Rickettsiae. Am J Trop Med Hyg. 2005;73(6):1083-5.

8. Parola P, Diatta G, Socolovschi C, Mediannikov O, Tall A, Bassene H, et al. Tick-borne relapsing fever borreliosis, rural Senegal. Emerg Infect Dis. 2011;17(5):883-5.

9. Vieth S, Drosten C, Lenz O, Vincent M, Omilabu S, Hass M, et al. RT-PCR assay for detection of Lassa virus and related Old World arenaviruses targeting the L gene. Trans R Soc Trop Med Hyg. 2007;101(12):1253-64.

10. Atkinson B, Chamberlain J, Dowall SD, Cook N, Bruce C, Hewson R. Rapid molecular detection of Lujo virus RNA. J Virol Methods. 2014;195:170-3.

11. Weidmann M, Avsic-Zupanc T, Bino S, Bouloy M, Burt F, Chinikar S, et al. Biosafety standards for working with Crimean-Congo hemorrhagic fever virus. J Gen Virol. 2016;97(11):2799-808.

12. Kafetzopoulou LE, Efthymiadis K, Lewandowski K, Crook A, Carter D, Osborne J, et al. Assessment of Metagenomic MinION and Illumina sequencing as an approach for the recovery of whole genome sequences of chikungunya and dengue viruses directly from clinical samples. *BioRxiv,* doi:101101/355560. 2018.

13. Kumar S, Stecher G, Li M, Knyaz C, Tamura K. MEGA X: Molecular Evolutionary Genetics Analysis across computing platforms. Mol Biol Evol. 2018;35:1547-9.
