# Supplementary material for: Detection of Crimean-Congo Haemorrhagic Fever cases in a severe undifferentiated febrile illness outbreak in the Federal Republic of Sudan: A retrospective epidemiological and diagnostic cohort study
Source: PLoS Negl Trop Dis. 2019 Jul 10;13(7):e0007571. doi: 10.1371/journal.pntd.0007571 (PMC6645580; doi:10.1371/journal.pntd.0007571)
Supplement: S2 Text — (DOCX) [file pntd.0007571.s002.docx]

**S2: STROBE Statement—Checklist of items that should be included in reports of *cohort studies***

|  | Item No | Recommendation |
| --- | --- | --- |
| **Title and abstract** | 1 | 1. Indicate the study’s design with a commonly used term in the title or the abstract  - Done in title: reference to cohort study |
|  |  | 1. Provide in the abstract an informative and balanced summary of what was done and what was found  - Done in abstract in section methodology and principal findings |
| Introduction | | |
| Background/rationale | 2 | Explain the scientific background and rationale for the investigation being reported   - Introduction: Paras 1 & 4- 6 |
| Objectives | 3 | State specific objectives, including any prespecified hypotheses   - Introduction: para 6 |
| Methods | | |
| Study design | 4 | Present key elements of study design early in the paper   - Main text methods: paras 1-5; - Abstract methods section |
| Setting | 5 | Describe the setting, locations, and relevant dates, including periods of recruitment, exposure, follow-up, and data collection   - Introduction: paras 4 & 5, Figure 2; - Methods para 1 |
| Participants | 6 | 1. Give the eligibility criteria, and the sources and methods of selection of participants. Describe methods of follow-up  - Methods: para 1; - Results paras 1 &2; Table 3 - No follow-up as study worked with stored diagnostic legacy samples |
|  |  | (*b*) For matched studies, give matching criteria and number of exposed and unexposed 🡺 n/a, unmatched study |
| Variables | 7 | Clearly define all outcomes, exposures, predictors, potential confounders, and effect modifiers. Give diagnostic criteria, if applicable   - Methods paras 2, 3, Figure 3, - Results: Table 3 & 4 - S1 Technical appendix; |
| Data sources/ measurement | 8* | For each variable of interest, give sources of data and details of methods of assessment (measurement). Describe comparability of assessment methods if there is more than one group   - Methods paras 1, 5; - Results: paras 2 & Table 3, - S1 Technical Annex for detailed laboratory and sequencing assessment measures. |
| Bias | 9 | Describe any efforts to address potential sources of bias   - Results: sensitivity analysis Table 3; Discussion: para 5 & 6 |
| Study size | 10 | Explain how the study size was arrived at   - Methods: para 1 |
| Quantitative variables | 11 | Explain how quantitative variables were handled in the analyses. If applicable, describe which groupings were chosen and why   - Methods : para 5 |
| Statistical methods | 12 | 1. Describe all statistical methods, including those used to control for confounding  - Methods : para 5 |
|  |  | 1. Describe any methods used to examine subgroups and interactions  - n/a |
|  |  | 1. Explain how missing data were addressed:  - Methods: para 5; - Results Table 3 &4 notes; paras 4-6, 10; - Discussion para 10 |
|  |  | (*d*) If applicable, explain how loss to follow-up was addressed |
|  |  | 1. Describe any sensitivity analyses  - Methods: para 5; - Results: Table 3 |
| Results | | |
| Participants | 13* | (a) Report numbers of individuals at each stage of study—eg numbers potentially eligible, examined for eligibility, confirmed eligible, included in the study, completing follow-up, and analysed |
|  |  | 1. Give reasons for non-participation at each stage  - Results: paras 1&2 |
|  |  | 1. Consider use of a flow diagram  - Flow diagram not considered necessary but can be added if reviewers prefer. |
| Descriptive data | 14* | 1. Give characteristics of study participants (eg demographic, clinical, social) and information on exposures and potential confounders  - Results: paras 3,4,6, Table 3 |
|  |  | 1. Indicate number of participants with missing data for each variable of interest  - Results: table 3 |
|  |  | 1. Summarise follow-up time (eg, average and total amount)  - No follow-up time: data derived from legacy samples |
| Outcome data | 15* | Report numbers of outcome events or summary measures over time   - Results: paras 5, 6, 7, 8, 9, Table 4 |
| Main results | 16 | 1. Give unadjusted estimates and, if applicable, confounder-adjusted estimates and their precision (eg, 95% confidence interval). Make clear which confounders were adjusted for and why they were included  - No adjustment for confounders: descriptive statistics only |
|  |  | 1. Report category boundaries when continuous variables were categorized  - Table 3 |
|  |  | 1. If relevant, consider translating estimates of relative risk into absolute risk for a meaningful time period  - n/a |
| Other analyses | 17 | Report other analyses done—eg analyses of subgroups and interactions, and sensitivity analyses   - Sensitivity analysis: Table 3 - Genetic sequencing: para 7, S1 Technical appendix |
| Discussion | | |
| Key results | 18 | Summarise key results with reference to study objectives   - Discussion: para 1 & 3 |
| Limitations | 19 | Discuss limitations of the study, taking into account sources of potential bias or imprecision. Discuss both direction and magnitude of any potential bias   - Discussion : paras 5 &6 |
| Interpretation | 20 | Give a cautious overall interpretation of results considering objectives, limitations, multiplicity of analyses, results from similar studies, and other relevant evidence   - Discussion all paras |
| Generalisability | 21 | Discuss the generalisability (external validity) of the study results   - Discussion: paras 6 &7 |
| Other information | | |
| Funding | 22 | Give the source of funding and the role of the funders for the present study and, if applicable, for the original study on which the present article is based   - This has been given in the PLOS submission process: it is UK Department of Health and Social Services Overseas Development Assistance funds via the UK Public Health Rapid Support Team research budget |

*Give information separately for exposed and unexposed groups.

**Note:** An Explanation and Elaboration article discusses each checklist item and gives methodological background and published examples of transparent reporting. The STROBE checklist is best used in conjunction with this article (freely available on the Web sites of PLoS Medicine at http://www.plosmedicine.org/, Annals of Internal Medicine at http://www.annals.org/, and Epidemiology at http://www.epidem.com/). Information on the STROBE Initiative is available at http://www.strobe-statement.org.
